# Péritonite par perforation grêlique secondaire à une arête de poisson

**DOI:** 10.11604/pamj.2013.15.107.3025

**Published:** 2013-07-23

**Authors:** Karim Ibn Majdoub Hassani, Imane Toughrai

**Affiliations:** 1 Faculté de médecine et de pharmacie de Fès, Université Sidi Mohammed Ben Abdellah, département de Chirurgie, CHU Hassan II Fès, BP: 1893 Km2.200, Route de Sidi Hrazem, Fez 30000, Maroc

**Keywords:** Péritonite, perforation grêlique, arête de poisson

## Image en médecine

Le diagnostic préopératoire d'une perforation grêlique secondaire à un corps étranger est extrêmement difficile. Ces perforations surviennent le plus souvent au niveau de la région iléocæcale et se présente sous forme de tableaux d'appendicite voir même de péritonite. Très rarement ces perforations peuvent évoluer spontanément vers la guérison. Nous présentant le cas d'une patiente âgée de 70ans, sans antécédents pathologiques notables admise aux urgences dans un tableau de douleur abdominales diffuses évoluant depuis deux jours, accompagné de quelques épisodes de vomissements, sans autres signes accompagnateurs. L'examen clinique retrouve un patient fébrile à 38,6°C, stable sur le plan hémodynamique avec à l'examen abdominal une défense abdominale généralisée. Le reste de l'examen clinique est normal par ailleurs. Le bilan biologique montre une hyperleucocytose à 12300 élément/mm3 et une CRP à 94. La radiographie d'abdomen sans préparation (ASP) est revenue sans particularité. On a complété par un scanner abdominal qui a objectivé un épaississement digestif grêlique localisé circonférentiel régulier et symétrique avec rehaussement de la muqueuse et ‘dème de la sous muqueuse au sein duquel on individualise un corps étranger (probablement arête de poisson) semblant traverser sa paroi avec un épanchement intra péritonéal de moyenne abondance. Le diagnostic d'une péritonite par perforation grêlique par corps étranger a été retenu. Nous avons décidé d'opérer la malade avec à l'exploration chirurgicale un épanchement péritonéal louche et une perforation grêlique punctiforme, on a réalisé l'extraction de l'arête de poisson à travers une petite entérotomie puis suture de la perforation et un lavage et drainage de la cavité péritonéale. Les suites opératoires étaient simples. La malade a été revue en consultation trois mois, puis six mois plus tard, l'examen clinique chez elle a été sans particularité.

**Figure 1:**
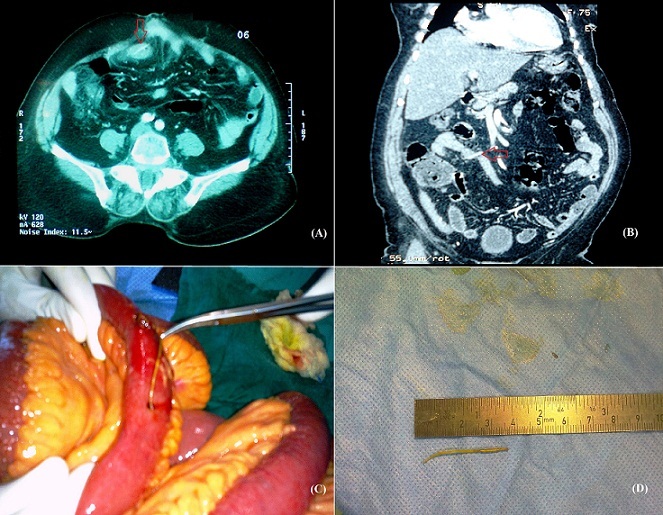
(A) coupe axiale (B) coupe coronale du scanner abdominal avec ingestion du produit de contraste montrant l’épaississement digestif grêlique localisé avec rehaussement de la muqueuse et l'arête de poisson qui traverse sa paroi; (C) extraction de l'arête de poisson à travers l'enterotomie; (D) l'arête de poisson

